# Rapid and Visual Identification of *Chlorophyllum molybdites* With Loop-Mediated Isothermal Amplification Method

**DOI:** 10.3389/fmicb.2021.638315

**Published:** 2021-03-18

**Authors:** Nan Wang, Zhiyong Zhao, Jie Gao, Enjing Tian, Wenjie Yu, Hui Li, Juan Zhang, Ruibin Xie, Xiaoyan Zhao, Ailiang Chen

**Affiliations:** ^1^Institute of Quality Standard and Testing Technology for Agro-Products, Key Laboratory of Agro-product Quality and Safety, Chinese Academy of Agricultural Sciences, Beijing, China; ^2^Institute for Agri-food Standards and Testing Technology, Shanghai Academy of Agricultural Sciences, Shanghai, China; ^3^Institute of Mycology, Engineering Research Center of Chinese Ministry of Education for Edible and Medicinal Fungi, Jilin Agricultural University, Jilin, China

**Keywords:** poisonous mushroom, loop-mediated isothermal amplification, *Chlorophyllum molybdites*, ITS, on-site rapid detection

## Abstract

*Chlorophyllum molybdites* is a kind of common poisonous mushroom in China that is widely distributed in different areas. Food poisoning caused by accidentally eating *C. molybdites* has become more frequent in recent years. In 2019, there were 55 food poisoning incidents caused by eating this mushroom in China. Mushroom poisoning continues to be a common health issue of global concern. When mushroom poisoning occurs, an effective, simple, and rapid detection method is required for accurate clinical treatment or forensic analysis. For the first time, we established a loop-mediated isothermal amplification (LAMP) assay for the visual detection of *C. molybdites*. A set of specific LAMP primers was designed, and the specificity was confirmed against 43 different mushroom species. The LAMP method could detect as low as 1 pg of genomic DNA. Boiled mushrooms and artificial gastric-digested mushroom samples were prepared to test the applicability of the method, and the results showed that as low as 1% *C. molybdites* in boiled and digested samples could be successfully detected. The LAMP method can also be completed within 45 min, and the reaction results could be directly observed based on a color change under daylight by the naked eye. Therefore, the LAMP assay established in this study provides an accurate, sensitive, rapid, and low-cost method for the detection of *C. molybdites*.

## Introduction

As a larger fungus, mushrooms are widely distributed worldwide with high nutritional value and pharmacological effects (Lei et al., 2020). According to statistics, there are currently more than 14,000 species of mushrooms in the world, and more than 3,800 species are known in China, of which approximately 480 are poisonous (Wu et al., 2019). Many edible mushrooms have similar morphological characteristics to poisonous mushrooms, and thus, it is difficult to distinguish poisonous mushrooms by the naked eye (Yang, 2013). In China, many food poisoning incidents are caused by poisonous mushrooms every year (Chen et al., 2014; Sun et al., 2018). According to statistics, there were 276 independent mushroom poisoning incidents in 2019, involving 769 patients from 17 provinces and 22 deaths, with an overall mortality rate of 2.86% (Haijiao et al., 2020). Among them, *Chlorophyllum molybdites* is a typical type of mushroom that is prone to be eaten by mistake. *C. molybdites* is widely distributed throughout the year in different regions of China, which has become the most poisonous mushrooms closest to humans (Soltaninejad, 2018). In 2018, 55 mushroom poisoning incidents caused by accidental eating of *C. molybdites* involving at last 133 patients were reported in China (Soltaninejad, 2018).

The mushroom poisoning incidents have become one of the serious food safety issues in China. For different types of poisonous mushrooms, they contain various toxins that result in different kinds of toxic symptoms. Mis-eating of *C. molybdites* mainly causes serious gastrointestinal discomfort that can last from 1 to 6 h after consumption. The toxin is a protein called molybdophyllysin (Yamada et al., 2012). If not treated in time, it will cause multiple organ failure and is life-threatening. Therefore, the rapid and accurate identification of mushroom species is necessary for proper clinical treatment. In food poisoning incidents caused by poisonous mushrooms, it has been found that mushroom samples lose their original morphological features after being cooked and digested. Therefore, identifying mushrooms by morphological identification methods is impossible. Various methods for mushroom species identification have been developed, in which the detection of mushroom toxins and DNA-based molecular biology methods are the most widely used (Li et al., 2016). With the discovery of various toxins in mushrooms, methods for identifying mushroom species by detecting toxins have been developed, such as chromatography, liquid chromatography diode array detection, inductively coupled plasma atomic emission spectrometry, and other technologies (Ahmed et al., 2010; Yao et al., 2018; Wurita et al., 2019; Numanoglu Cevik, 2020). However, most of these methods require expensive instruments, a long detection cycle, and high detection costs, which are not suitable for application in primary regions. The rapid detection of toxins represented by lateral flow immunoassays has the advantages of low cost, rapid detection, and intuitive results, but the toxin antibodies are not stable and are difficult to prepare, and the sensitivity and specificity need to be improved for practical applications, especially with respect to processed products (Tripathi et al., 2018). As the basis of the genetic material of organisms, nucleic acid would not be affected by age, season, developmental stage, and environment factors, and has a high thermal stability that could not be interfered by alterations during processing (Hebert et al., 2003; Garnica et al., 2016). With the development of polymerase chain reaction (PCR), a variety of diagnostic methods have been derived, such as real-time PCR, polymerase chain reaction–restriction fragment length polymorphism (PCR-RFLP), and DNA barcoding (Sugano et al., 2017; Kondo et al., 2019; Wu et al., 2019). However, these methods usually rely on expensive instruments and are time-consuming, which were technically challenging to achieve on-site rapid detection. Therefore, the development of simple, efficient, accurate, and sensitive mushroom species identification methods would be helpful for timely symptomatic treatment in the early onset of poisoning.

Isothermal amplification methods have been widely used for species identification of pathogenic fungi in recent years (He et al., 2019; Qiu et al., 2019; Bodulev and Sakharov, 2020). Among them, loop-mediated isothermal amplification (LAMP) is the most widely used one, which has received increasing attention (Lee, 2017). LAMP was first proposed by Notomi et al. (2000). The LAMP reaction contains a set of four to six specific primers, which can quickly identify six to eight regions on the target sequences and bind to strand displacement active DNA polymerase. LAMP reaction can be carried out at a constant temperature (60–65°C), generating a vast number of amplicons (Lee, 2017). There are various detection methods for LAMP products; among them, the colorimetric method is easily used in on-site detection. The reaction result of the colorimetric method is visible to the naked eye, and it can be realized by only relying on a water bath without expensive equipment (Wang et al., 2014). After more than 20 years of development, LAMP has been widely used in the field of pathogenic microorganism rapid text (Lv et al., 2020), clinical disease diagnosis (Wang W.T. et al., 2019), food hygiene inspection (Zhu and Wang, 2018), and environmental monitoring (Becherer et al., 2020), in view of its efficient, specific, and sensitive detection capabilities, as well as a wide range of applications and the capacity of this technology to be performed outside of the routine laboratory environment. In terms of mushroom species identification, Vaagt et al. (2013) first applied the LAMP-based technique to the detection of *Amanita phalloides*. He et al. (2019) established the LAMP method for 10 poisonous *Amanita* detections. To date, there is no report on the detection and identification of *C. molybdites*.

In this study, the LAMP assay based on the colorimetric method targeting the internal transcribed spacer (ITS) was established for the rapid detection of *C. molybdites* from edible mushrooms with similar shapes and other common toxic mushrooms. In addition, considering the practical application of this method, mushroom processing and digestion in the human body were simulated. The LAMP detection method of *C. molybdites* established in this study is of great significance for the rapid identification of the toxic mushrooms in poisoning events, the targeted treatment after poisoning, and the prevention and diagnosis of poisoning.

## Materials and Methods

### Sample Collection

**FIGURE 1:**
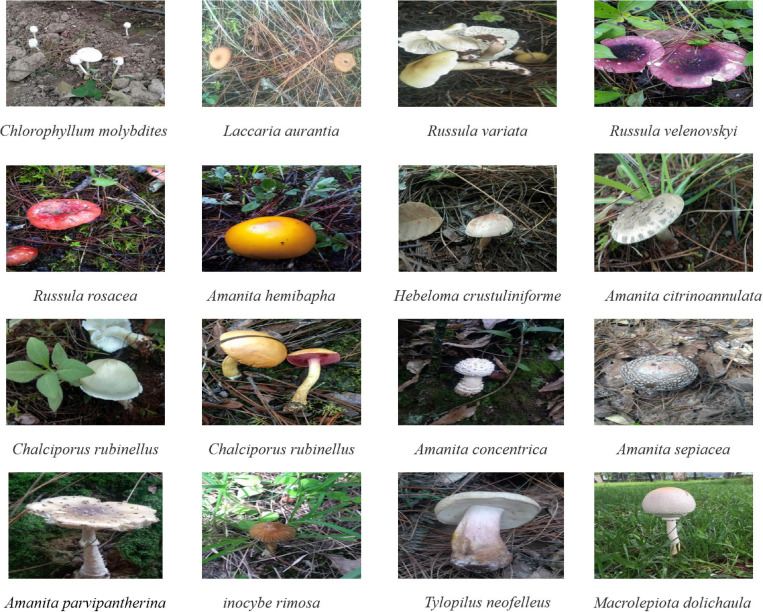
Some photographs of mushroom species collected in this study. Among them, *Chlorophyllum molybdites* was collected in Yunnan, China (24°52′N and 102°49′E), and the other mushrooms were collected from Shanghai, China (31°11′N and 121°29′E). The picture of *C. molybdites* was taken by ZZ, and the remaining 15 photos of mushrooms were taken by LZ.

A total of 44 mushroom species were collected in this study, including 15 poisonous mushrooms (Figure 1). These mushrooms mainly include edible fungi similar to *C. molybdites* (e.g., *Macrolepiota*), common poisonous and edible poisonous fungi in China. Among them, the *C. molybdites* sample was collected from a vineyard in Shanghai, China, in June 2020; and the remaining 43 mushrooms were collected from Lijiang, Yunnan, China, in July to September 2020. All samples were collected with the entire sporocarps and were initially identified using morphological characteristics. Furthermore, DNA barcoding methods based on ITS genes were carried out to cross-confirm the identities of the above 44 mushrooms. All samples were stored at −20°C for further analysis.

### DNA Extraction, PCR, and Sanger Sequencing

All samples were rapidly dried by liquid nitrogen and then fully ground into powder. According to the manufacturer’s instructions, the extraction of total genomic DNA was performed by a DNA secure plant kit (TIANGEN, China). The concentration and quality of DNA were measured by a Nanodrop 2000 ultramicro-spectrophotometer (Thermo Fisher Scientific, United States) and then diluted to 10 ng/μL for further study. A universal primer of ITS4 and ITS5 (White et al., 1989) was used for PCR amplification of the 44 mushrooms in a PCR thermal cycler (Applied Biosystems, United States). The PCR mixtures contained 12.5 μL of 2 × Taq PCR Master Mix, 0.3 μL of each primer, 3 μL of DNA, and 6.8 μL of ddH_2_O in a total volume of 25 μL. Primer amplification of the ITS fragments was conducted in the following conditions: initial denaturation at 94°C for 5 min, followed by 25 cycles of 94°C for 30 s, 58°C for 30 s, and 72°C for 30 s, and a final extension step at 72°C for 10 min. Amplified PCR products were evaluated by electrophoresis on a 1.5% agarose gel and then sent to Beijing Sangon Biological Technology (China) for Sanger sequencing. The SeqMan program in the DNASTAR software was used to check and splice the two-way sequencing results to remove the low-quality region. The spliced sequences were used as queries for BLAST analysis in National Center for Biotechnology Information (NCBI^1^) based on sequence similarity and comprehensive score. Finally, we submitted the obtained sequence to the GenBank database (accession numbers MW192451–MW192494).

### LAMP Primers Design

Internal transcribed spacer gene sequences were chosen as the amplification target to design LAMP-specific primers of *C. molybdites*. Eleven available ITS sequences of *C. molybdites* were downloaded from the NCBI GenBank database (accession numbers: KM609396.1, MK541937.1, MK541933.1, MK541934.1, MK541935.1, MK541936.1, MK541940.1, MK560059.1, MK541938.1, MK541941.1, MK541939.1). Then multiple sequence alignments were performed to seek the intraspecific conserved interval of *C. molybdites*, which served as the primer design region. To ensure the specificity of primers, the ITS sequences of the other 17 species of *Chlorophyllum* (*C. neomastoideum*, *C. arizonicum*, *C. sphaerosporum*, *C. agaricoides*, *C. alborubescens*, *C. brunneum*, *C. demangei*, *C. globosum*, *C. hortense*, *C. levantinum*, *C. lusitanicum*, *C. nothorachodes*, *C. olivieri*, *C. palaeotropicum*, *C. pseudoglobosum*, *C. rhacodes*, and *C subrhacodes*) and 43 mushrooms collected in this study were downloaded to align with *C. molybdites* using the MEGA software. Specific LAMP primers were designed in the interspecific differentiated and intraspecific conservative intervals.

**FIGURE 2:**
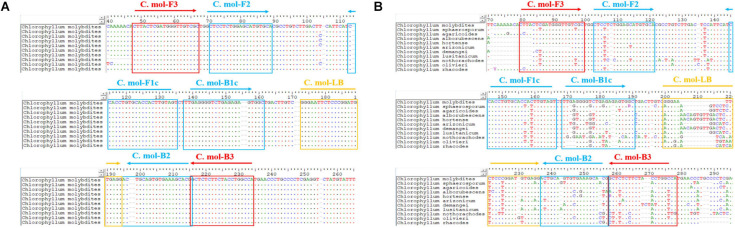
Positions of the LAMP primer set of *Chlorophyllum molybdites* designed in the region of the ITS gene. **(A)** Multiple sequences alignment of homologous species of *C. molybdites*. **(B)** Multiple sequences alignment of related species of *C. molybdites* (not all shown in the figure).

A set of *C. molybdites* specific LAMP primer was designed by using GLAPD^2^ (Jia et al., 2019). For the multiple primer sets output by GLAPD, a theoretical verification for specificity of the LAMP primers was carried out by Primer-BLAST^3^ in NCBI. Then, we determined whether there are primer dimers, hairpins, false priming, and cross dimer using the Primer Premier version 5 software. Finally, validation experiments were conducted on each specific and good quality primer set to confirm the amplification efficiency and specificity. The primers with the highest amplification efficiency, good method stability, and specificity were selected as the LAMP-specific primers for this study. The selected LAMP primers contained two outer primers (C. mol-F3 and C. mol-B3), two inner primers (C. mol-FIP primer consisted of the complementary sequences of F1c and F2 and C. mol-BIP primer consisted of B1c and B2), and a loop primer (C. mol-LB). The position and sequences of the *C. molybdites* specific LAMP primer are shown in Figure 2 and Table 1.

**TABLE 1:** The LAMP primer set of *Chlorophyllum molybdites* used in this study.

Primers	Sequence (5′-3′)
C. mol-F3	CTTACTCGATGGGTTGTCGC
C. mol-FIP	ACTACAAGTGGTGCACAGGTGGCTCCTCTGGAGCATGTGCA
C. mol-BIP	TTGAGGGGTCTGAGAGAGTGGCCGTGCTTTCACACTGCAGT
C. mol-B3	TGGCCAGGTAGAAGAGAGC
C. mol-LB	GGGAATTCTCCCGGATGTGAGG

### LAMP Reaction and Specificity Analysis

The two pairs of outer primers, two inner primers, and loop primers were pre-diluted with ddH_2_O to 200, 50, and 200 μM in concentration, respectively, and then they were mixed in a ratio of 3:10:96 (V/V/V). LAMP reaction was conducted via a PCR thermal cycler (Applied Biosystems, United States) in a 10-μL volume, which contained 5 μL of WarmStart Colorimetric LAMP 2X Master Mix (New England Biolabs, United States), 1.09 μL of primer mixtures, 2 μL of the DNA template, and replenishing ddH_2_O to 10 μL. Amplification of the primers for the ITS fragment was carried out under the temperature of 65°C for 45 min. After the reaction phase, we placed the reaction tubes on a white paper and then judged the reaction results according to the color change. The results of the positive LAMP were determined by direct visual observation of the change in color from pink to yellow in visible light.

To assess the specificity of newly designed LAMP primers, genomic DNA from 43 mushrooms collected in this study were tested by the LAMP assay. If only the color of positive control (*C. molybdites*) turned to yellow, while the negative and blank control remained pink, the specificity of the primer set was good.

### LAMP Method Suitability Analysis

To further verify the applicability of the LAMP method established in this study, we simulated the course of mushroom processing and body digestion process. A series of mushroom samples, including boiled and digested samples, were subjected to analysis by LAMP.

Mushrooms of *C. molybdites* and *Pleurotus eryngii* were mixed, respectively, in the final mass ratios of 1:1, 1:10, and 1:100 (∼100 mg total mass) using the obtained powders. Prepared mushrooms of single *C. molybdites* and mixtures were boiled in water for 10 min at 100°C, respectively. DNA extraction and LAMP reaction were performed as described above.

Artificial gastric fluid was made up of 0.05 g potassium chloride, 0.42 g sodium chloride, and 0.32 g pepsin in 100 mL water, and then the pH was adjusted to 2.0 with 2 mol/L hydrochloric acid (Minekus et al., 2014). Boiled mushrooms of single *C. molybdites* and mixtures (*C. molybdites* and *P. eryngii* with the final mass ratios of 1:1, 1:10, and 1:100, respectively) were then incubated in artificial gastric fluid for 4 h at 37°C, respectively. DNA extraction and LAMP reaction were performed as described above. In addition, the existence of human genes in the digestive tract, as well as other potential pollutants, such as the enterobacteria group, was considered. In order to further ensure the accuracy of the LAMP detection method established in this study, we conducted a specific verification experiment of LAMP primers on human saliva.

## Results

### Species Identification of Sanger Sequencing

A total of 44 samples of genomic DNA were obtained. The DNA concentration of all samples was higher than 10 ng/μL, and the ratio of 260/280 was between 1.8 and 2.2, which was enough to meet the requirements of the subsequent study. With the ITS gene as the amplification target, the true identities of 44 collected mushroom samples were further verified by Sanger sequencing technology. The PCR amplification and sequencing success rate of all samples was 100%. The obtained sequences were blasted with the GenBank database from NCBI. The comparison results showed that ITS sequence similarity of all 44 species was ≥98% against the reference sequences, and the identification results confirmed the initial taxonomic assessment (Table 2). Subsequently, the ITS gene sequencing sequences of these 44 mushroom samples were uploaded to NCBI.

**TABLE 2:** Species identification of 44 mushrooms based on Sanger sequencing.

Sample ID	Results of ITS (sequence similarity)	GenBank accession no.
M1	*Chlorophyllum molybdites* (100%)	MW192451
M2	*Suillus bovinus* (99.4%)	MW192452
M3	*Gymnopus subnudus* (99.73%)	MW192453
M4	*Panaeolus subbalteatus* (99.36%)	MW192454
M5	*Leucoagaricus rubrotinctus* (99.70%)	MW192455
M6	*Macrolepiota dolichaula* (99.72%)	MW192456
M7	*Lactarius subbrevipes* (99.81%)	MW192457
M8	*Laccaria aurantia* (99.85%)	MW192458
M9	*Amanita griseofolia* (100%)	MW192459
M10	*Rhizocybe alba* (98.32%)	MW192460
M11	*Russula variata* (99.33%)	MW192461
M12	*Russula senecis* (98.57%)	MW192462
M13	*Amanita hemibapha* (100%)	MW192463
M14	*Boletus kauffmanii* (99.32%)	MW192464
M15	*Tylopilus neofelleus* (100%)	MW192465
M16	*Russula rosacea* (99.68%)	MW192466
M17	*Butyriboletus yicibus* (99.87)	MW192467
M18	*Russula velenovskyi* (100%)	MW192468
M19	*Hydnellum caeruleum* (99.73%)	MW192469
M20	*Tricholoma saponaceum* (98.35%)	MW192470
M21	*Inocybe mixtilis* (100%)	MW192471
M22	*Hydnellum concrescens* (98.52%)	MW192472
M23	*Amanita spissacea* (99.48%)	MW192473
M24	*Tricholoma albobrunneum* (100%)	MW192474
M25	*Pleurotus eryngii* (100%)	MW192475
M26	*Flammulina filiformis* (99.83%)	MW192476
M27	*Lentinula edodes* (100%)	MW192477
M28	*Hypsizygus marmoreus* (100%)	MW192478
M29	*Tricholoma olivaceoluteolum* (100%)	MW192479
M30	*Amanita citrinoannulata* (100%)	MW192480
M31	*Russula crustosa* (99.70%)	MW192481
M32	*Hebeloma crustuliniforme* (99.71%)	MW192482
M33	*Tricholoma imbricatum* (99.86%)	MW192483
M34	*Amanita parvipantherina* (100%)	MW192484
M35	*Amanita verrucosivolva* (100%)	MW192485
M36	*Tricholoma matsutake* (100%)	MW192486
M37	*Amanita concentrica* (99.86%)	MW192487
M38	*Amanita sepiacea* (98.90%)	MW192488
M39	*Pleurotus ostreatus* (100%)	MW192489
M40	*Tylopilus microsporus* (100%)	MW192490
M41	*Inocybe rimosa* (98.91%)	MW192491
M42	*Amanita pseudovaginata* (100%)	MW192492
M43	*Russula sanguinea* (99.31%)	MW192493
M44	*Gymnopus dryophilus* (100%)	MW537049

### Specificity and Sensitivity Analysis

**FIGURE 3:**
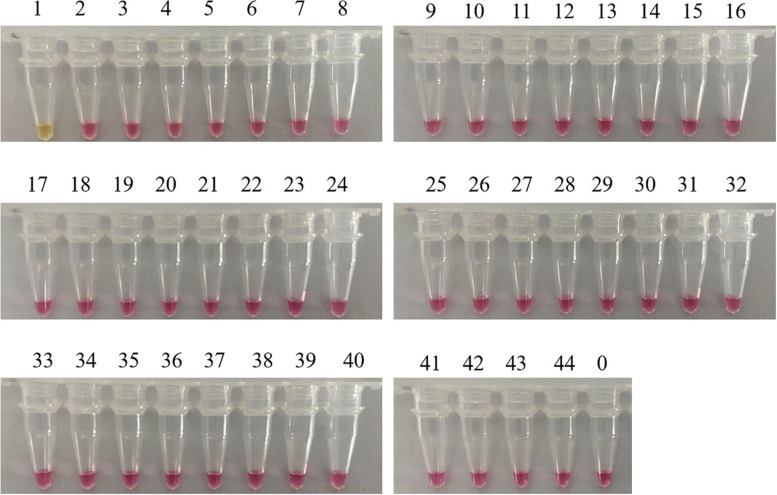
Specificity test of the LAMP primer set for *Chlorophyllum molybdites* designed in this study. The set of primer was amplified for the detection of *C. molybdites* and 43 other non-target species. 1: *C. molybdites*; 2: *Suillus bovinus*; 3: *Gymnopus subnudus*; 4: *Panaeolus subbalteatus*; 5: *Leucoagaricus rubrotinctus*; 6: *Macrolepiota dolichaula*; 7: *Lactarius subbrevipes*; 8: *Laccaria aurantia*; 9: *Amanita griseofolia*; 10: *Rhizocybe alba*; 11: *Russula variata*; 12: *Russula senecis*; 13: *Amanita hemibapha*; 14: *Boletus kauffmanii*; 15: *Tylopilus neofelleus*; 16: *Russula rosacea*; 17: *Butyriboletus yicibus*; 18: *Russula velenovskyi*; 19: *Hydnellum caeruleum*; 20: *Tricholoma saponaceum*; 21: *Inocybe mixtilis*; 22: *Hydnellum concrescens*; 23: *Amanita spissacea*; 24: *Tricholoma albobrunneum*; 25: *Pleurotus eryngii*; 26: *Flammulina filiformis*; 27: *Lentinula edodes*; 28: *Hypsizygus marmoreus*; 29: *Tricholoma olivaceoluteolum*; 30: *Amanita citrinoannulata*; 31: *Russula crustosa*; 32: *Hebeloma crustuliniforme*; 33: *Tricholoma imbricatum*; 34: *Amanita parvipantherina*; 35: *Amanita verrucosivolva*; 36: *A. verrucosivolva*; 37: *Amanita concentrica*; 38: *Amanita sepiacea*; 39: *Pleurotus ostreatus*; 40: *Tylopilus microsporus*; 41: *Inocybe rimosa*; 42: *Amanita pseudovaginata*; 43: *Russula sanguinea*; 44: *Gymnopus dryophilus*; 0: ddH_2_O.

A reliable species-specific LAMP amplification primer set should display a high degree of specificity among various species and should be highly conserved within the same species. Genomic DNA from 43 mushrooms was used to evaluate the specificity of the *C. molybdites* LAMP primer set designed in this study. For the LAMP assay, as shown in Figure 3, the color change (pink to yellow) only occurred in *C. molybdites*, indicating that the primer amplification was successful, whereas for the 43 negative controls and the blank control, no cross-reaction was detected and the color of the reaction tubes remained pink. Thus, we could prove that the LAMP primer set designed in this study is *C. molybdites*-specific.

**FIGURE 4:**
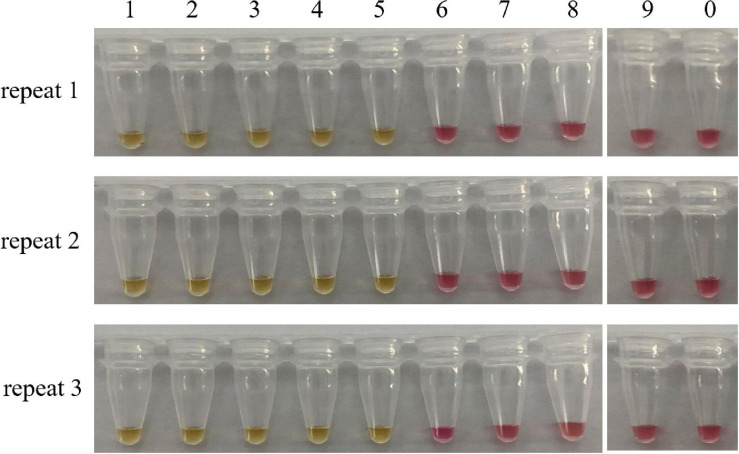
Sensitivity test of the LAMP assay. Minimum amount of detectable DNA of the LAMP method established in this study was evaluated by using a series of *C. molybdites* DNA dilutions. For each reaction tube, we carried out three replicates. The color changed to yellow indicated positive amplification. 1: 10 ng; 2: 1 ng; 3: 0.1 ng; 4: 0.01 ng; 5: 1 pg; 6: 0.1 pg; 7: 0.01 pg; 8: 1 fg; 9: 0.1 fg; 0: ddH_2_O.

Sensitivity refers to the minimum amount of DNA that can be detected. For this purpose, we prepared a series of 10-fold dilutions of the genomic DNA of *C. molybdites*, ranging from 10 ng to 0.1 fg. According to the color change of the reaction tube, the limit of detection (LOD) of the LAMP method was 1 pg (Figure 4). It is much lower than many traditional detection methods (Epis et al., 2010; Praphruet and Charerntantanakul, 2014). Simultaneously, we compared the LAMP sensitivity of *C. molybdites* without a loop primer. In the absence of a loop primer, the LOD of the LAMP method was 10 pg, indicating that the loop primer could enhance sensitivity (Supplementary Figure 1).

### Method Applicability Analysis

**FIGURE 5:**
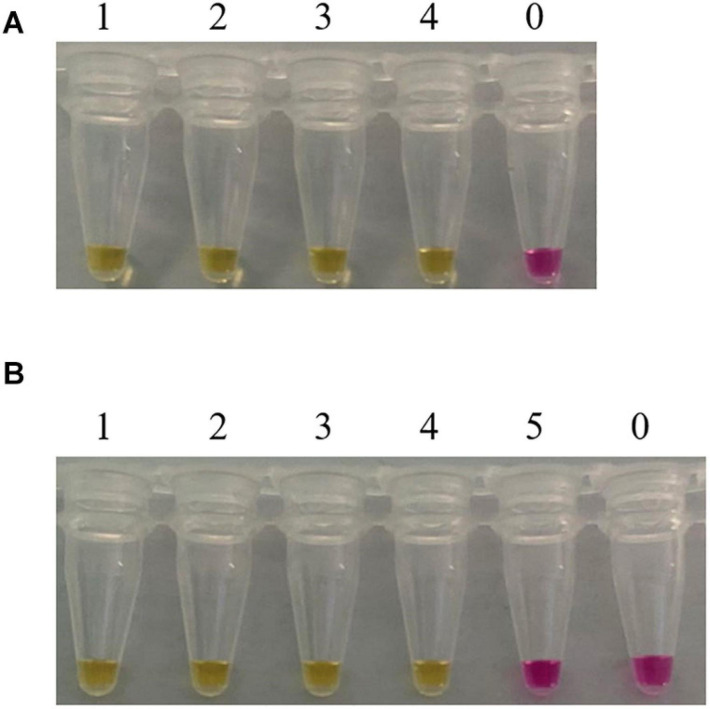
Suitability assessment of the LAMP assay. Feasibility analysis of the LAMP method in on-site detection was evaluated by simulating the course of processing and digestion of mushrooms. **(A)** LAMP assay used for the detection of boiled *Chlorophyllum molybdites.* 1: *C. molybdites*; 2: 50% *C. molybdites* + 50% *Pleurotus eryngii*; 3: 10% *C. molybdites* + 90% *P. eryngii*; 4: 1% *C. molybdites* + 99% *P. eryngii*; 0: ddH_2_O. **(B)** LAMP assay used for the detection of digested *C. molybdites* after boiled. 1: *C. molybdites*; 2: 50% *C. molybdites* + 50% *P. eryngii*; 3: 10% *C. molybdites* + 90% *P. eryngii*; 4: 1% *C. molybdites* + 99% *P. eryngii*; 5: human saliva; 0: ddH_2_O.

Consider that, in practical applications, mushrooms are usually processed before being consumed by people. Furthermore, in clinical practice, we usually test the vomit or feces of patients with mushroom poisoning. When mushrooms are accidentally ingested and entered the stomach, their DNA would be further degraded by the digestive juices. In order to evaluate the feasibility of our method, LAMP assays were carried out in boiled and digested samples. As shown in Figure 5, the LAMP primer set designed in this study detected the *C. molybdites* from both single and mixture samples that had been boiled and digested, as well as in the mixtures with a *C. molybdites* content as low as 1%. The blank controls were not reacted, and the LAMP primers reacted negatively to human saliva. In conclusion, the LAMP method could be successfully applied for the detection of processed and digested *C. molybdites* in both single and mixtures.

## Discussion

Mushroom gatherers may mistakenly identify mushrooms from the species of *C. molybdites* as edible, thus causing food poisoning. In this study, we established a LAMP method that is relatively simple and field-adaptable.

One of the challenges in establishing an efficient LAMP detection assay is the selection of species-specific amplification targets (Tomita et al., 2008). Mitochondrial DNA has been widely studied in species for the selection of target genes based on its multiple copies, matrilineal inheritance, and rapid evolution rate (Dulay et al., 2020; Mesic et al., 2020). Among these, the ITS gene has been proven to have high intraspecific differences and low interspecies variation. Schoch et al. (2012) tested six target regions (SSU, LSU, ITS, RPB1, RPB2, and MCM7) commonly used in the molecular identification of fungi. They proposed the use of ITS genes as the official fungal amplification target. This proposal was supported by the scientific community and later used as the Barcode of Life^4^ by the Consortium. ITS gene fragments are commonly used in the detection and identification of mushroom species (Garnica et al., 2016; Badotti et al., 2017; Wang S. et al., 2019). A rich and complete database is the basis for the design of specific primers. Fungi are a group of organisms that show great diversity in morphology, life cycle, and ecology; the current research on fungi is very limited. In molecular biology studies on fungal species, a large number of species are only represented by a single strain or specimen, and thus, nucleotide variation data for within-species and close-source species comparisons are lacking (Xu, 2016). Information on such variations is essential to distinguish closely related species (Xu, 2016). As one of the fungi with large species abundance, mushrooms are also faced with the problem of lack of genetic information. Therefore, we first examined the integrity and availability of nucleic acid sequences of *Chlorophyllum* before designing the specific *C. molybdites* LAMP primer. According to the literature, there are about 20 species of *Chlorophyllum* recorded in the world (Vellinga, 2002; Ge et al., 2018). We searched the ITS sequences of these species through NCBI, and all the species were successfully searched. Therefore, in the present study, we selected the ITS gene as the molecular marker to design species-specific LAMP primer sets.

In the selection of binding sites for LAMP primers, sufficient numbers of mismatches with non-target species should be confirmed. For example, rainbow trout (*Oncorhynchus mykiss*) LAMP primers designed by Xiong et al. (2020) had a total of 43 mismatch sites against their near-source species (*Salmo salar*, *Oncorhynchus gorbuscha*, *Oncorhynchus tshawytscha*, *Oncorhynchus keta*, *Oncorhynchus masou*, and *Oncorhynchus nerka*), resulting in high specificity for the rainbow trout. The primer set designed in this study had several mismatches against other species of *Chlorophyllum* at the 3′ end of C. mol-F3/B3 and the 5′ end of C. mol-F1c/B1c regions (Figure 1), demonstrating the high specificity of the *C. molybdites* primer set.

The sensitivity of LAMP detection is usually 10–100 times higher than that of conventional PCR assays (Wang et al., 2015). The method proposed in the present study proved to have high sensitivity and could detect 1 pg of *C. molybdites* DNA. This conclusion was confirmed by multiple independent repeated experiments (Figure 4). In related studies, the LAMP detection method established by Chen Zuohong’s research team from Hunan Normal University for lethal *Amanita* species had a detection limit of 10 pg (He et al., 2019). The sensitivity of the LAMP assay might be affected by many factors. It has been reported that the addition of loop primers could increase the sensitivity and specificity of the LAMP reaction (Nagamine et al., 2002). Nagamine et al. (2002) found that significant amplification of 10^3^ lambda DNA could be observed with loop primers. While removing loop primers, the signal was dispersed among 10^3^ copies within 52–92 min, which proved that significant DNA amplification required at least 10^4^ copies, indicating a higher sensitivity of the LAMP reaction with loop primers. The detection limit of *C. molybdites* in our study also confirmed this. In addition, some researchers found that the amount of DNA templates larger than 200 ng had an inhibitory effect on the LAMP reaction (Njiru et al., 2008). In the current study, when the content of *C. molybdites* DNA added to the reaction system was 100 ng, the LAMP reaction was inhibited, and the color of the final product was an intermediate color of pink and yellow instead of yellow, which was consistent with the findings of Njiru et al. (2008) (results were not shown in Figure 4). This is presumably due to the complexity of the mushroom matrix, which contains many polysaccharides and polyphenols (Abubakar et al., 2018). Polyphenols can be oxidized after a long time, with irreversibly bound proteins and nucleic acids forming high molecular weight complexes (Newbury and Possingham, 1977). Polysaccharides can hinder the activity of polymerase and inhibit the amplification reaction of LAMP (Fang et al., 1992). Therefore, during detection, attention should be paid to controlling the content of the DNA template in the reaction system.

Visualization of LAMP amplification results is more conducive to rapid field detection. A variety of LAMP rapid visual inspection methods have been developed in recent years. Among them, the most commonly used methods are turbidity detection and color change (Tomita et al., 2008; Denschlag et al., 2013). The turbidity detection method is simple and cost-saving. However, this method has low detection sensitivity. When the concentration of the amplified product is low, the result is not always visible (Njiru et al., 2008). The detection method based on a color change has proven to be an effective alternative method. Many metal ion indicators, such as hydroxyl naphthol blue (HNB) and calcein, are used as indicative dyes for LAMP amplification (Pang et al., 2019; Chen et al., 2020; Wigmann et al., 2020). Whether the reaction has occurred can be determined according to the color change in the tubes. The reaction results are visible to the naked eye, and furthermore, there is no need to open the tubes during the reaction process, which can effectively avoid aerosol contamination and the probability of false positives (Goto et al., 2009). However, the LAMP amplification products are not obvious in terms of color changes with the HNB and calcein dyes. The WarmStart^®^ Colorimetric LAMP 2X Master Mix (New England Biolabs, United States) used in this study contains a visible pH indicator. During the LAMP reaction, the pH of the reaction solution tends to decrease because of the production of protons and the subsequent enhancement of DNA polymerase activity, such that the color of the solution changes from pink to yellow. The color change is obvious, and the reaction result is visible to the naked eye under daylight conditions, which is consistent with the results of Daskou et al. (2019). In addition, considering that researchers are affected by subjective preference, we put the reaction tube on white paper after amplification and used the white color of the paper as a control to ensure that the difference in color between positive and negative tubes became more pronounced. Furthermore, double-blind experiments were carried out in the laboratory, and all observers had no disagreement with the test results. The LAMP visual detection method established in this study will provide ideas for the further development of microfluidic chips and integrated on-site rapid detection methods.

For mushroom poisoning incidents, in the examination of clinical patients and forensic investigation, the detection of vomit, feces, and stomach contents is required. In order to ensure the feasibility of this method, we simulated the process of mushroom processing and digestion in human gastric juice. In this study, we successfully detected *C. molybdites* in the mixed mushrooms digested with artificial gastric juice. High temperature, pressure, and the digestion of gastric juice may cause some degree of genomic DNA degradation and fragmentation. Previous studies have confirmed that DNA degradation would not affect the detection of fragments shorter than 300 bp (Goldstein and Desalle, 2003). Gausterer et al. (2012) used direct PCR to investigate the stomach contents in a forensic case and successfully detected the digested yew plant. Others had proved that DNA was still present in traditional food processing styles, and it could be amplified in dried mushrooms and even be incubated by gastric juices (Gausterer et al., 2014). The LAMP method for *C. molybdites* detection established in this study had the same conclusion. In conclusion, our study provided an evidence that the LAMP assay could be used for the rapid and sensitive detection of *C. molybdites* in various matrices, including raw, boiled, and digested mushrooms with single or mixed species.

## Conclusion

According to the results of this present study, molecular biology methods focused on nucleic acid are of great necessity to detect mushrooms of *C. molybdites*. More specifically, the LAMP method targeting the mitochondrial ITS gene was established for the on-site rapid detection of *C. molybdites*. High specificity and sensitivity (the LOD of detectable DNA was 1 pg) for the LAMP method were confirmed in the present study. Finally, the established method was successfully applied to the boiled and digested mushroom mixtures. These characteristics indicated that the LAMP assay for species-specific detection was useful for identifying *C. molybdites* in clinical treatment and forensic analysis.

## Data Availability Statement

The original contributions presented in the study are included in the article/Supplementary Material, further inquiries can be directed to the corresponding author/s.

## Author Contributions

AC conceived of and designed the experiments. ZZ and XZ provided some *Chlorophyllum molybdites* materials and identified the species. ET completed the morphological identification of the mushroom samples. HL and JZ completed the analysis of ITS DNA sequences of all mushrooms. NW, JG, WY, and RX carried out the LAMP assay. NW and AC wrote the manuscript. All authors contributed to the article and approved the submitted version.

## Conflict of Interest

The authors declare that the research was conducted in the absence of any commercial or financial relationships that could be construed as a potential conflict of interest.

## Footnotes

1 https://www.ncbi.nlm.nih.gov/

2 http://cgm.sjtu.edu.cn/GLAPD/online/

3 https://www.ncbi.nlm.nih.gov/tools/primer-blast/

4 http://barcodinglife.org/

## Supplementary Material

Supplementary Figure 1 Sensitivity test of the *Chlorophyllum molybdites* LAMP assay without a loop primer. Minimum amount of detectable DNA of the LAMP method established in this study was evaluated by using a series of *C. molybdites* DNA dilutions. The color changing to yellow indicated positive amplification. 1: 10 ng; 2: 1 ng; 3: 0.1 ng; 4: 0.01 ng; 5: 1 pg; 6: 0.1 pg; 7: 0.01 pg; 8: 1 fg; 9: 0.1 fg; 0: ddH_2_O.
