# Supplementary figures and images for: Rapid and Visual Identification of Chlorophyllum molybdites With Loop-Mediated Isothermal Amplification Method
Source: Front Microbiol. 2021 Mar 18;12:638315. doi: 10.3389/fmicb.2021.638315 (PMC8013719; doi:10.3389/fmicb.2021.638315)

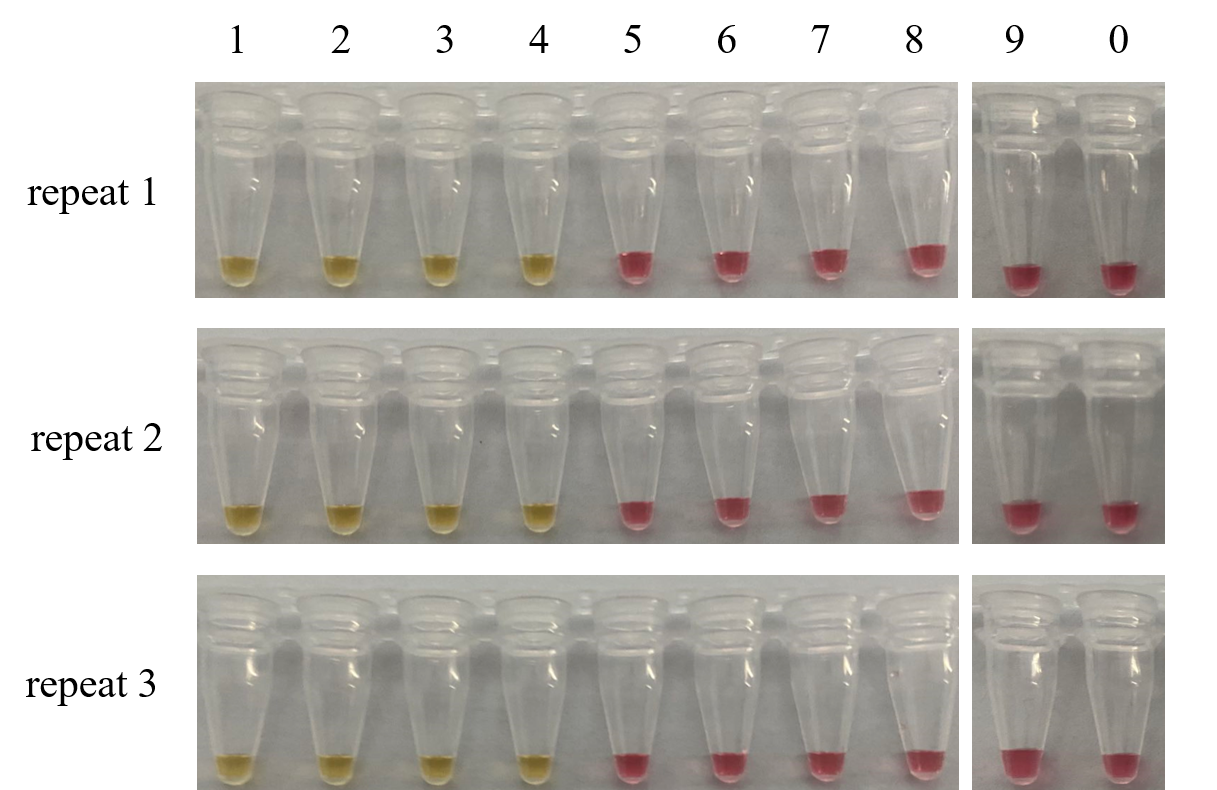

Supplement: Supplementary Figure 1 — Sensitivity test of the Chlorophyllum molybdites LAMP assay without a loop primer. Minimum amount of detectable DNA of the LAMP method established in this study was evaluated by using a series of C. molybdites DNA dilutions. The color changing to yellow indicated positive amplification. 1: 10 ng; 2: 1 ng; 3: 0.1 ng; 4: 0.01 ng; 5: 1 pg; 6: 0.1 pg; 7: 0.01 pg; 8: 1 fg; 9: 0.1 fg; 0: ddH2O. [file Image_1.TIF]
